# Overview of 3D-Printed Silica Glass

**DOI:** 10.3390/mi13010081

**Published:** 2022-01-03

**Authors:** Han Zhang, Long Huang, Mingyue Tan, Shaoqing Zhao, Hua Liu, Zifeng Lu, Jinhuan Li, Zhongzhu Liang

**Affiliations:** Center for Advanced Optoelectronic Functional Materials Research, and Key Laboratory for UV Emitting Materials and Technology of Ministry of Education, National Demonstration Center for Experimental Physics Education, Northeast Normal University, 5268 Renmin Street, Changchun 130024, China; z15678103856@163.com (H.Z.); huangl284@nenu.edu.cn (L.H.); tanmy697@nenu.edu.cn (M.T.); juggerzhao@yeah.net (S.Z.); luzf934@nenu.edu.cn (Z.L.); lijh248@nenu.edu.cn (J.L.); liangzz@nenu.edu.cn (Z.L.)

**Keywords:** 3D printing, silica glass, additive manufacturing

## Abstract

Not satisfied with the current stage of the extensive research on 3D printing technology for polymers and metals, researchers are searching for more innovative 3D printing technologies for glass fabrication in what has become the latest trend of interest. The traditional glass manufacturing process requires complex high-temperature melting and casting processes, which presents a great challenge to the fabrication of arbitrarily complex glass devices. The emergence of 3D printing technology provides a good solution. This paper reviews the recent advances in glass 3D printing, describes the history and development of related technologies, and lists popular applications of 3D printing for glass preparation. This review compares the advantages and disadvantages of various processing methods, summarizes the problems encountered in the process of technology application, and proposes the corresponding solutions to select the most appropriate preparation method in practical applications. The application of additive manufacturing in glass fabrication is in its infancy but has great potential. Based on this view, the methods for glass preparation with 3D printing technology are expected to achieve both high-speed and high-precision fabrication.

## 1. Introduction

Additive manufacturing (AM) is defined as “the process of manufacturing objects layer by layer by connecting materials through 3D model data”, which is also known as the additive manufacturing process and free-form fabrication [1]. Additive manufacturing was first invented and developed in the 1980s and, since then, the technology has gained traction thanks to its rapid, mold-less molding, as well as its relatively short cycle of manufacturing and the ability to replicate accurately. Over the next few years, the technologies associated with additive manufacturing (AM) will rapidly evolve and transform into a more comprehensive manufacturing approach. In addition, additive manufacturing has an extremely high material utilization rate compared with traditional manufacturing processes, which helps to achieve satisfactory geometric precision [2]. All these unique features have led to a greater diversity of materials produced using additive manufacturing techniques, including even polymers [3], metals [4,5], soft matter [6], and nanocomposites. The great complexity of geometries in additive manufacturing has been widely used in aerospace, energy storage, and optical precision instruments [7], and it has even demonstrated some exceptional capabilities and potential for rapid response to public health emergencies [8].

Currently, additive manufacturing technology is also extensively applied to the manufacturing of polymer and metal objects [9,10,11,12]. The application of glass materials to 3D printing technology is not yet widespread due to their high melting point and high hardness characteristics. Glass materials have shown great applicability in optical devices given their many important assets, such as unparalleled optical transparency and excellent physical as well as chemical properties. However, the traditional manufacturing methods for glass are time consuming and labor intensive, and glass cannot be precisely molded. The process of molding glass is still being explored. Despite the hardships, 3D printing technology with photocuring and high-temperature sintering and other processing methods are now available to process glass materials [13,14,15,16,17,18,19,20]. The 3D printing glass field has been widely concerned with improvement and has been increasingly recognized year by year, and it is now understood to be very valuable for research in the future.

Glass technologies for 3D printing can be divided into several categories according to the printing method and the form of pre-treatment for the raw materials. These categories include powder-based, photopolymerization-based, and material extrusion-based 3D printing technology, as shown in Table 1. Among them, fused deposition modeling (FDM), based on material extrusion (MEX), and selective laser sintering/melting (SLS/SLM), based on powder, usually require strict processing conditions and are therefore less suitable for laboratory processing. The most promising processing technologies are stereolithography (SLA), digital light processing (DLP), two-photon polymerization (TPP), sheet lamination (SL), which is based on photopolymerization, and DIW, based on MEX.

So far, time-consuming and expensive customization, low dimensional precision, and rough surface finish are the main challenges in the process of glass fabrication through 3D printing technology [21,22,23]. The purpose of this review is to discuss the latest state of 3D printing technologies for fabricating glass. The paper provides an overview of the development of popular technologies, describes general methods for preparing glass with 3D printing technology, gives representative examples of applications, and compares important aspects of glass 3D printing technology. Finally, future challenges and opportunities for glass 3D printing technology are envisioned and discussed in order to achieve wider applications.

## 2. Classification of 3D Printing Glass Technologies

In recent years, more than 50 types of 3D printing technologies based on different principles have been developed according to the different requirements of materials, printing speed, and printing precision [24]. Only the following technologies have been reported for glass fabrication: SLS/SLM, FDM, SLA, DLP, TPP, and DIW, as well as SL. Among them, although FDM is mostly used to prepare glass crafts due to the characteristics of the processing technology, it cannot be widely used in the optical field [25,26,27]. While the SLS/SLM technique provides good control of the crystal structure and achieves nearly 100% material utilization [24], the processing process generates vapor bubbles, which limits the optical transparency of the glass. In addition, Luo et al. proposed a method to produce silica glass using the fiber feeding method. However, although the initially prepared samples had some transparency, a tedious polishing process was required to achieve high transparency properties [28,29,30]. This has limited the development of this technology in the optical field.

**Figure 1:**
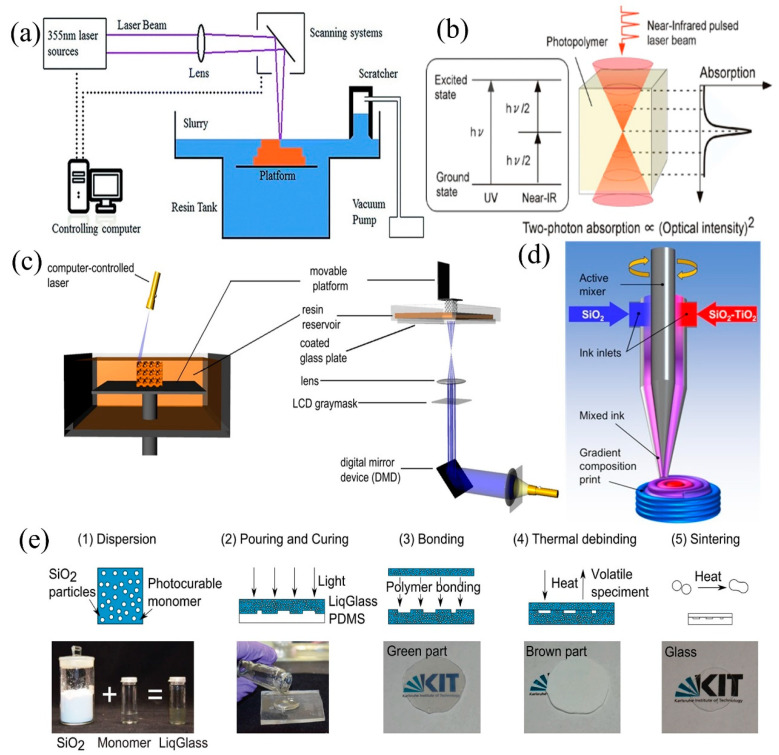
Schemes for (**a**) SLA [39], (**b**) TPP, (**c**) DLP, (**d**) DIW [14], and (**e**) sheet lamination. (**1**) (**2**) (**3**) (**4**) and (**5**) in (**e**) represent each step of the sheet lamination process. (**b**) Reprinted with permission from ref. [40]. (**c**) Reprinted with permission from ref. [41]. (**e**) Reprinted with permission from ref. [33].

Therefore, in terms of the efficiency and practicality of glass microstructure preparation, the 3D printing methods that can be used to prepare glass microstructures are mainly of two main types: (1) four methods based on photopolymerization with “liquid glass” materials [13,31,32,33,34,35] and (2) DIW based on material extrusion. All the schematics of the technologies is shown in Figure 1. Samples fabricated using these methods require a reasonable heat treatment process to form silica glass [36,37,38]. These methods are described below, focusing on the printing resolution and speed, the requirements for the material ratio that serve as guidelines for selecting the appropriate method, and techniques for improving the printing quality and efficiency. 

### 2.1. Micro-Stereolithography (μ-SLA)

Micro-stereolithography can be divided into direct laser writing (DLW) technology, which is based on spot exposure, and DLP, which is based on area exposure according to the exposure mode. For DLW, the UV exposure-based technology is usually called stereolithography (SLA), and the femtosecond laser-based technology is called two-photon polymerization (TPP).

#### 2.1.1. Stereolithography (SLA)

SLA, based on photopolymerization, was first patented and commercialized by C. Hull in 1986 [42], and the schematic diagram is shown in Figure 1a. The first process is to design a 3D model using CAD, then to use a discrete program to slice the model and design the scan path, using the CNC (computer numerical control) equipment to control the laser movement [24,43,44,45]. When the object is processed, the laser spot selectively cures the “liquid glass” photopolymerizable material, accumulating the stack layer by layer until the final 3D object is completed. Because the process uses computerized 3D drawing software to design the internal and external geometry of the structure to be printed, it could be freed from the constraints of traditional molds and be used for the molding and fabrication of many complex components.

SLA was initially used in the additive manufacturing of polymer materials [42]. With the development of more advanced technology, E. Fantino et al. also applied SLA technology to the additive manufacturing of ceramic and glass materials [46,47,48,49,50]. The resolution of SLA depends on the size of the laser spot and generally reaches the tens of microns, which is still far from the standard of micro-optical devices. For this case, the TPP process has been developed.

#### 2.1.2. Two-Photon Polymerization (TPP)

By replacing the UV laser in SLA with a femtosecond laser, TPP could be achieved. TPP is a micro- and nanofabrication technique proposed by Maruo et al. in 1997 [51]. The schematic diagram for TPP is illustrated in Figure 1b. In this process, a photopolymer absorbs two near-infrared photons simultaneously in a single quantum event whose collective energy corresponds to the UV region of the spectrum. The rate of two-photon absorption is proportional to the square of the light intensity so that the near-infrared light is strongly absorbed only at the focal point within the photopolymer. The quadratic intensity dependence of two-photon absorption allows for the confinement of the solidification to submicron dimensions, which are beyond the diffraction limit of light [40]. Two-photon polymerization is capable of fabricating 3D micro- and nanostructures at a higher resolution than other processes. Moreover, the lateral resolution of TPP below 100 nm has been demonstrated [2,52,53,54,55]. Currently, the use of TPP technology for the additive manufacturing of glass materials is a new trend [56,57]. 

The common problem with DLW technology is the low processing efficiency caused by spot exposure, which usually means it takes hours or even days to process millimeter-level objects using SLA or TPP. Under these circumstances, DLP with the improved light source emerged [58,59].

#### 2.1.3. Digital Light Processing (DLP) 

DLP technology using an area exposure dates back to 1996, when it initially used liquid crystal displays (LCD) as dynamic displays, though these were replaced in 2001 by digital micromirror devices (DMD). A DMD is a chip that consists of rectangular arrays of hundreds of thousands of micromirrors corresponding to the pixels on the image plane to be displayed [60,61,62,63]. The ultra-fast light switching and integral projection greatly reduce the time required for 3D printing and allow for feature resolutions of a few microns.

Limited by the size of the DMD, with DLP, it is difficult to achieve uniform projection on large surfaces, so the technology is mostly used for the processing of microstructures. Due to the demand for high manufacturing precision and the increase in machinable materials, DLP is considered to be one of the most powerful and versatile processes, and is more suitable for the preparation of micro-optical devices [64,65]. Figure 1c demonstrates the schematic diagram for DLP. The devices can be divided into two types according to their structures: bottom-up (left) and top-down (right) systems. The bottom-up system does not break due to gravity and the release force (the pulling force on the inverted equipment when the sample comes out of the bottom of the resin tank), which makes it suitable for massive production, but it is more likely to generate air bubbles and affect the quality of the molded parts. Although the inverted processing technique is affected by gravity and the release force, this processing system has a closed optical path and is relatively safe. The resin flows naturally at the bottom of the resin tank without the influence of air bubbles or uneven liquid levels [41,66]. The height of printing is not affected by the depth of the resin, so it can print larger objects with a handful of materials, which makes it more suitable for laboratory design and development.

DLP has received widespread attention because of its printing speed and its ability to achieve micron-level molding precision. In 2017, Frederik Kotz et al. were able to achieve the 3D printing of glass parts by adding SiO_2_ nanopowder to the photocuring resin in combination with heat treatment, which also sets a precedent in the field of glass devices using DLP [35].

In the 3D printing of glass, the three technologies (SLA, DLP, and TPP) of micro-stereolithography (μ-SLA) have something in common. The 3D printing of glass is achieved by adding silica nanoparticles to the polymerization of monomers. The SiO_2_ nanoparticles are well dispersed in the polymer monomer and then become suspended with the aid of the necessary surfactants and additives. During the process of 3D printing, the SiO_2_ nanoparticles are uniformly surrounded by the cross-linked organic network before being polymerized, forming each layer into a pre-designed shape until the entire structure is built up [2]. In order to form silica glasses with high density, the printed structure must be processed with suitable heat treatment. The heat treatment process is usually divided into two steps: low-temperature debinding and high-temperature sintering.

### 2.2. Direct Ink Writing (DIW)

The above-mentioned processes are suitable for the preparation of single-material objects but cannot realize the preparation of multi-material structures. Therefore, the DIW printing process has been developed. DIW is a technique for manufacturing objects by extruding material from a nozzle and depositing it on a selected area. The core components of the technology are the ink material ratios as well as the deposition nozzles, and the technology was first proposed by Cesarano in 1997 [2]. Direct ink writing (DIW) is an extrusion-based additive manufacturing method, which has found a large number of applications in the field of material and tissue engineering. In the process of DIW printing, liquid-phase “ink” is dispensed out of nozzles under controlled flow rates, and then deposited layer by layer along pre-designed paths until the 3D object is fully fabricated [67,68]. The sprayed ink is rapidly cured due to evaporative phase change, or gelation, as shown in Figure 1d. 

The process of DIW is relatively simple, allows for inexpensive manufacture, and could be used with a wide range of materials, making it suitable for desktop-level printing. It is also possible to manufacture objects consisting of multiple materials by switching the nozzle [69,70,71,72,73]. Furthermore, DIW technology has also been successfully applied by Dylla-Spears’ team to the manufacture of transparent silica glass [15,74].

Using DIW technology for 3D printing glass also requires the addition of a suitable amount of silica nanoparticles to the ink of DIW, while the shaping and heat-treatment process is also needed. The difference with the μ-SLA process for printing glass is that the heat treatment parameters need to be altered somewhat due to the different forming methods of the DIW technology.

### 2.3. Sheet Lamination (SL)

Sheet lamination is also based on the use of “liquid glass” as well as photocuring, and is a technique for replicating structures that uses a soft mold made of PDMS [33,75]. This technology allows for the use of “liquid glass” with a higher SiO_2_ content because it does not need to take the severe diffuse reflections that occur during SLA/DLP printing into account. In SL, the photocured object is transformed into dense, high-quality glass through heat treatment. The technique is highly efficient, and can accurately replicate objects ranging from the microscale to the centimeter scale. Since the preparation process does not require hazardous chemicals or cleanroom facilities, the manufacturing process is cost-effective and could be performed in any laboratory [33]. The scheme for sheet lamination is shown in Figure 1e.

The process of 3D printing glass using the SL process is pretty much the same as with μ-SLA, with the main difference being that SL-printed objects are usually larger in size and require more suitable heat treatment parameters.

## 3. Applications of 3D Printing Glass Technologies

In the actual processing of 3D glass materials, different forming methods give the forming structure different characteristics. For example, DLW technology is widely used in the laboratory; DIW technology makes it easier to achieve material ratio changing and realize multi-material structure processing; sheet lamination facilitates rolling lamination, so it is suitable for preparing large-sized structures. The unique advantages of these technologies have led to the different applications of 3D printing glass technology.

### 3.1. Applications of SLA Technology for Printing Glass

The creation of glass devices using the SLA technique is achieved by uniformly dispersing fumed silica nanopowder with a particle size of about 40 nm in a polymer monomer for photocuring, followed by heat treatment [35]. Due to the poor precision and slow processing speed of the SLA technique, the preparation of glass devices using this technique has rarely been reported, and only Liu et al. have used this technique to prepare rare-earth-doped optical glasses, demonstrating some promising applications [39,76].

**Figure 2:**
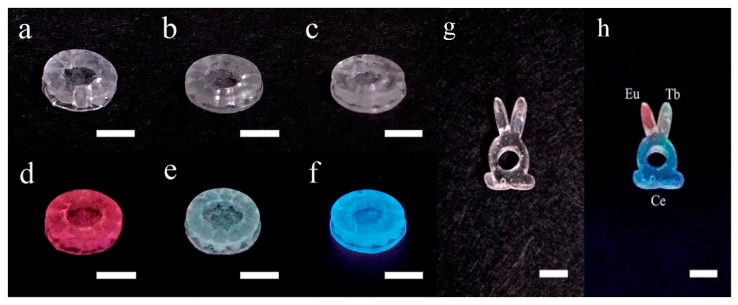
Photos of the 3D-printed silica glass doped with (**a**) Eu^3+^, (**b**) Tb^3+^, and (**c**) Ce^3+^ ions, and their luminescent performance under a 254 nm UV lamp are shown in (**d**–**f**). Photos of selected-area-doped silica glass under (**g**) natural light and (**h**) 254 nm UV radiation. Scale bar: 5 mm [75].

Liu et al. doped glass with rare earth ions (e.g., Eu^3+^, Tb^3+^, and Ce^3+^) using a 3D printing technique, and these emitted red, green, and blue light under the excitation of a 254 nm UV lamp, as shown in Figure 2a–f. Moreover, the results imply that the shape and function of individual glass devices can be designed through 3D printing. In addition, different rare earth ions were doped at different locations in the glass through selected doping, and Eu^3+^ and Tb^3+^, as well as Ce^3+^, were doped in different areas of a silica glass prepared using the SLA technique. Under natural light, the glass does not show the differences between the regions (Figure 2g), while under the UV lamp at 254 nm, the different regions can have different fluorescence emissions and realize the colored light emission, as shown in the right of Figure 2h. The emergence of this technology has turned the realization of different optical properties in the same glass into a reality, which has promoted the development of the field of optoelectronic materials and devices, changed the fabrication of optoelectronic devices, and provided new ideas for the preparation of optoelectronic materials.

### 3.2. Applications of TPP Technology for Printing Glass

**Figure 3:**
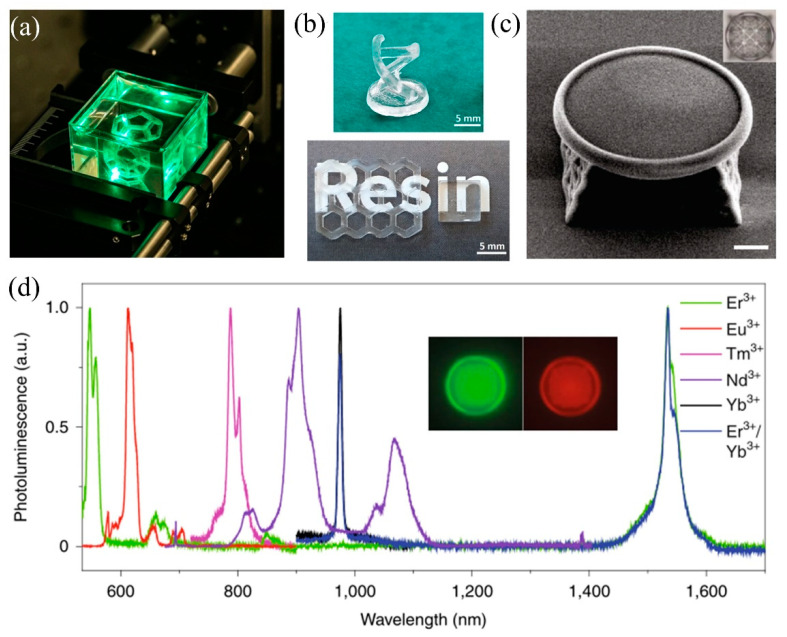
(**a**) 3D printing of a complex part in the experimental set-up. (**b**) Examples of printed and sintered silica glass parts. (**c**) SEM image of the printed micro-toroid optical resonator (scale bar, 10 μm); inset: optical image from the top. (**d**) Photoluminescence of Er^3+^-, Eu^3+^-, Tm^3+^-, Nd^3+^-, and Yb^3+^-doped and Er^3+^/Yb^3+^ (1:1)-co-doped silica crystal in the visible to near-infrared range; inset: the Er^3+^-doped micro-toroid optical resonator under 495 nm (left) and 592 nm (right) excitation and observed at 519 nm (left) and 614 nm (right) using a fluorescence microscope. (**a**,**b**) Reprinted with permission from ref. [56] © The Optical Society. (**c**,**d**) Reprinted with permission from ref. [57].

TPP technology could solve the above problems and can directly produce 3D nanostructures whose spatial resolution is well beyond the diffraction limit. In 2021, Thomas Doualle et al. applied two-photon polymerization to 3D-printed glass by adding an appropriate photoinitiator to “liquid glass”, successfully relying on a laser-based process of multi-photon-induced polymerization to produce complex three-dimensional (3D) glass parts [56]. The object in the process of 3D printing is presented in Figure 3a, and the successfully printed glass devices are shown in Figure 3b [56]. This work pioneered the use of TPP technology for the additive manufacturing of glass materials.

Moreover, Wen Xiewen et al. have developed a TPP 3D printing technique using PEG-functionalized colloidal silica NPs that can be highly loaded [57]. Using 3D printing and post-sintering techniques, high-quality 3D silica structures with arbitrary shapes of amorphous glass or polycrystalline square quartz shapes could be created at resolutions below 200 nm. Figure 3c illustrates the SEM image of the printed micro-toroid optical resonator. Figure 3d shows that each photoluminescence peak matches the atomic transition line of a single rare-earth element, confirming the flexibility of the method in the doping/co-doping of rare-earth elements and revealing the potential of constructing passive and active integrated micro-photonic chips using 3D printing.

### 3.3. Applications of DLP Technology for Printing Glass

DLP printing technology has a faster printing speed compared to SLA and TPP technology and shows great advantages when printing larger-sized objects, and has become a widely chosen and efficient additive manufacturing method in laboratories [77,78,79]. The existing literature reports that this technique is mostly used for the preparation of glass devices, including optical fibers, optical glasses, etc., and could also be combined with lamination processes to produce hollow microfluidic channels.

**Figure 4:**
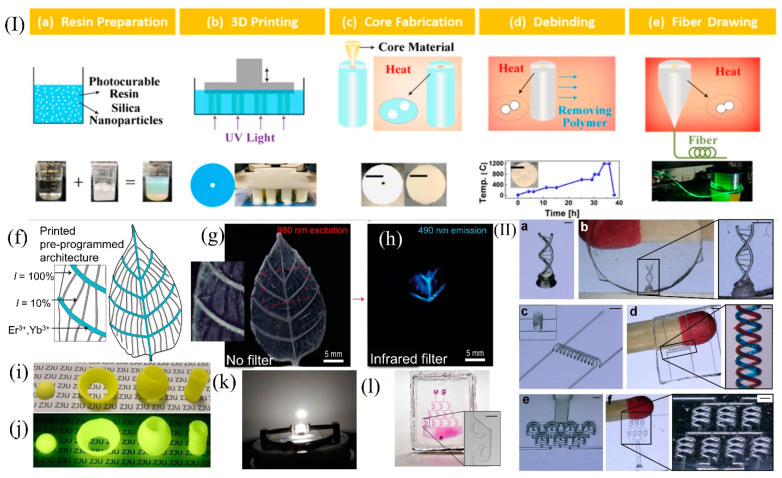
(**I**) (**a**–**e**) Fabrication process of the step-index silica optical fiber using 3D printing. The patterns of primary and secondary veins are shown in (**f**,**g**). The area covered with doped glass under infrared light radiation shows intense blue light, as shown in (**h**). (**i**) Examples of printed and sintered YAG:Ce-PiSG (scale bar, 1 cm) and (**j**) the same objects under UV radiation [16]. (**k**) Lighted WLED device in the darkness [16]. (**l**) Microlithography of an exemplary microfluidic chip (inset scale bar, 200 μm) (**II**). (**a**) Polymeric DNA double helix (scale: 500 μm). (**b**) Inverse structure in fused silica glass (scale: 400 μm). (**c**) Intertwined spirals (scale: 900 μm). (**d**) Resulting intertwined microfluidic spiral channels in fused silica glass with a channel width of 74 μm. The channels were filled with dyes (see inset, scale: 140 μm). (**e**) Polymeric microstructures of an out-of-plane mixer structure (scale: 600 μm). (**f**) Microfluidic mixer structure in fused silica glass with a channel width of 74 μm (scale bar: 280 μm) [81]. (**I**) (**a**–**e**) Reprinted with permission from ref. [34] © The Optical Society. (**f**–**h**) Reprinted with permission from ref. [80]. (**l**) Reprinted with permission from ref. [35].

YuShi Chu et al. were the first to use the DLP process to easily prepare the core and cladding of optical fibers by controlling the size and uniformity of silica nanoparticles, and successfully drew the fibers [34]. The processing procedure of the printing method used for step refractive index fibers is illustrated in Figure 4I(a–e). The advantage of using DLP photocuring technology is that different refractive indices of the cladding and core can be easily achieved by changing the raw material ratio to ensure light propagation. After the raw materials for the fiber cores are rationed, they are poured into the printed preforms and undergo further thermal curing, as shown in Figure 4I(c). The prepared preforms were subsequently thermally degreased to remove polymers and other impurities, as depicted in Figure 4I(d). After debinding, the preform blanks are inserted into Heraeus F300 quartz tubes for support, and fibers with a length of L = 2.3 km are drawn directly on a commercial drawing tower, as shown in Figure 4I(e). Related tests have shown that the optical fibers prepared through 3D printing have preliminary optical properties.

The technology of 3D-printed glass has great value in optoelectronics as well. David G. Moore et al. [80] realized the 3D printing of glass leaves by regulating the light intensity to verify the accuracy of DLP technology. The rare-earth metals, Er^3+^ and Yb^3+^, were also directionally doped during the printing process, and the resulting 3D objects were subsequently heat-treated to form silica glass. The final glass leaf has a unique appearance when illuminated with a collimated beam of 980 nm infrared light. Photon up-conversion at the lanthanide-containing voxels turns the incident infrared radiation into blue light, making the primary veins of the glass leaf stand out colorfully in the otherwise transparent and translucent background pattern. The whole process is illustrated in Figure 4I(f–h). Compared with the previously mentioned SLA technique for preparing rare-earth-doped phosphors, the DLP technique makes it easier to achieve light intensity tuning and enables the more precise realization of locally controllable optical properties.

Moreover, Dao Zhang et al. used modern 3D printing technology to fabricate YAG:Ce-PiSG devices for white light illumination by combining the fabrication process of YAG:Ce-PISG [16]. The application and performance characterization of white LEDs with laser illumination was also performed, achieving >90% internal quantum efficiency and demonstrating device application, making the free-form manufacturing of fully inorganic color converters a reality and facilitating product design and small volume production, as labeled in Figure 4I(i–k).

The DLP process has also been greatly applied in the field of microfluidics, and Figure 4I(l) shows a microfluidic glass chip constructed through microlithography, which initially highlights the application of the process in the field of microfluidics [35]. The applications can go much further by selecting the suitable material and using DLP technology for stencil structures or immiscible phases, and combing the object with lamination technology to emboss the stencil into the glass structure. Through dissolution, etching, or combustion, which involve the continuous removal of the sacrificial template, and then through the heat treatment process of degreasing, sintering can finally achieve the preparation of complex three-dimensional microstructures in the glass [82,83,84,85]. For example, in 2019, Frederik Kotz et al. proposed applying the sacrificial template method to prepare hollow microstructures in silica glass. PEGDA was used as a template, and various shapes of microfluidic channels have been prepared. All the microfluidic channels are presented in Figure 4II(a–f). Using this method, microfluidic channel structures with low aspect ratios and high aspect ratios could be easily fabricated, and the prepared 3D structure channels could be reproduced with high fidelity and without distortion [81].

The application of the DLP process to the preparation of optical fibers and microfluidic channels simplifies the preparation process and enables the application of 3D printing in the field of optics and microfluidic channels [86,87,88,89]. DLP has been widely applied and reported, and its advantages are outstanding, although the disadvantage that the preparation of multi-material structures is not able to be realized in one step is still obvious.

### 3.4. Applications of DIW Technology for Printing Glass

**Figure 5:**
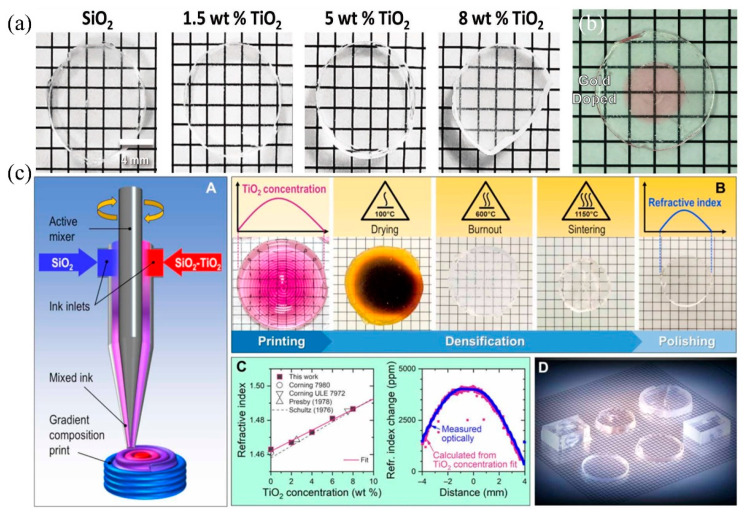
(**a**) Related glass sample with a typical polish, which is fully dense and prepared through DIW. (**b**) Two nozzles containing gold and non-gold silica ink were used to print the core-shell structure. (**c**) Process and samples of additive manufacturing of gradient index (GRIN) silica-titania glass via DIW [14]. (**a**) Reprinted with permission from ref. [74]. (**b**) Reprinted with permission from ref. [13].

One of the major advantages of DIW is the ability to change the material of the ink easily according to the requirements. For example, Joel F et al. used sol-gel-derived inks to prepare homogeneous, optical quality silica and silica-titanium dioxide glass monoliths through DIW [74]. Figure 5a presents glasses prepared using DIW, with the use of the inks obtained through the sol-gel method, and this glass has core-shell TiO_2_-SiO_2_ nanoparticles with different titanium percentages for glass refractive index tuning.

Another advantage of DIW is that the slurry nozzles can be modified directionally when needed, for example, by adding a second dispensing nozzle to the mechanical bench where multiple materials could be combined. Using this method, a core-shell structure of silicon oxide and silica ink mixed with gold nanoparticles can be formed, as illustrated in Figure 5b, where the apparent red color is resulted from the surface plasmon resonance of gold nanoparticles at 528 nm due to their strong absorption [13].

In addition, multi-material blanks containing silica nanoparticles and different concentrations of titanium dioxide as the refractive index modulating dopants were prepared at the Lawrence Livermore National Laboratory. The sample fabrication process using the DIW technique is depicted in A in Figure 5c [14]. The optical glasses with different sizes, shapes, and functions in D in Figure 5c were obtained using the heat treatment process presented in B in Figure 5c. C in Figure 5c illustrates the refractive index spatial distribution of the glasses. This shows that the method can be used to achieve a variety of conventional and unconventional optical functions in flat glass components without surface curvature. It is promising to expand the capabilities of 3D printing technology and open the door to the preparation of previously undiscovered functional materials in glass, which may have applications in optics, sensing, packaging, and microfluidic systems.

DIW technology provides a broad idea for the preparation of plasma-resonant multi-component and multifunctional glasses, and the ability to easily adjust the material ratios also highlights the application potential for the preparation of multi-component, multifunctional objects.

### 3.5. Applications of SL Technology for Printing Glass

Although the technologies of DLP and DIW are mature enough, the objects fabricated through DLP have an obvious “step effect”, while in DIW, it is difficult to complete the preparation of microstructures. These disadvantages seriously hinder their applications. Sheet lamination can solve these problems well.

**Figure 6:**
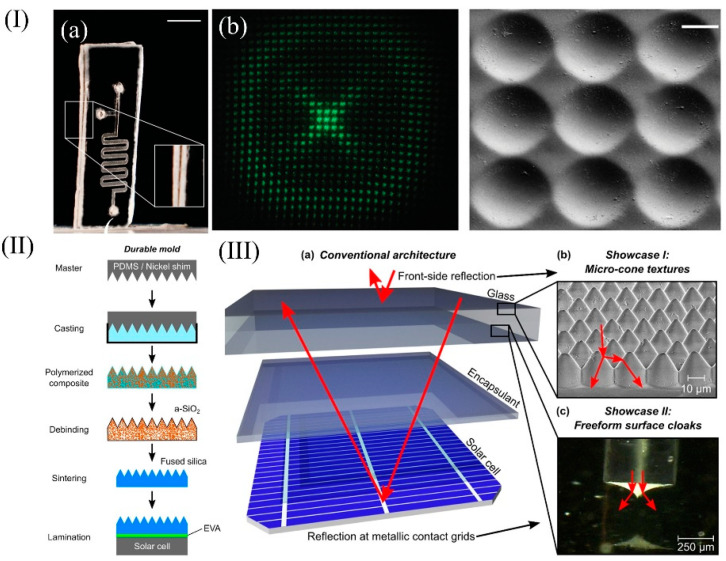
(**I**) (**a**) A microfluidic glass chip made using sheet lamination (scale bar: 4 mm). (**b**) Optical projection pattern obtained from a replicated micro-optical lens array made using sheet lamination with SEM image (scale bar: 15μm). (**II**) The processes of the sheet lamination. (**III**) (**a**) Schematic of the c-Si solar module architecture. (**b**) SEM image of the front-side reflection obtained by texturing the front cover glass and (**c**) reflection of metallic front contacts obtained by embedding free-form surface (FFS) cloaks into the front cover glass. (**I**) Reprinted with permission from ref. [33]. (**II**,**III**) Reprinted with permission from ref. [90].

The sheet lamination technique, also known as soft replication, usually uses PDMS as a template to obtain the target structure with a reverse mold, and then heat treatment is adopted to obtain the glass microstructure [31,33]. For example, the unique process has applications in the field of microfluidic chips, such as the microfluidic glass chip illustrated in Figure 6I(a), which consists of a two-layer structure that can be easily obtained. Smooth hollow channels are also obtained. In addition to this, the micro-lens array prepared using the sheet lamination technique demonstrates good integrity, which is illustrated in Figure 6I(b) [33].

Malte Langenhorst et al. applied the lamination technology to photovoltaics, applying the optical concept to the conventional architecture of encapsulated solar modules [90]. The flow chart is labeled in Figure 6(II), which is also a common process for lamination technology and, subsequently, it is only necessary to prepare the template according to the requirements and optimize the ratio and heat treatment process. The adoption of lamination technology marks the next milestone in the development of FFS cloaks that are compatible with the conventional solar module architecture outlined in Figure 6III(a). This technology enables the embedding of FFS cloaks into fused silica glass slides, creating a functionalized front cover glass for solar modules. The front cover glass with an embedded FFS cloak fabricated using sheet lamination technology will greatly improve its robustness and durability. Figure 6III(b) shows an SEM image of the front cover glass, and a close-up view of the air voids due to the embedded FFS cloak is presented in the microscopic image in Figure 6III(c). This image shows the excellent quality of the liquid glass technique by which FFS can be transferred into fused silica glass. The combination of “liquid glass” and sheet lamination technology to prepare the fused silica front cover glass reduced the frontal reflection loss by 80% and achieved an invisibility efficiency of 88%. Moreover, due to the presence of microstructures, the whole structure has a strong hydrophilic behavior, which may eventually be applied for self-cleaning purposes [91,92,93].

Using lamination technology, fused silica glass could be processed similarly to a polymer. This method combines the advantages of polymer processing with the excellent material properties of fused silica glass to form a new glass manufacturing method that is more efficient compared to traditional glass processing. The use of sheet lamination fills in the gaps between the manufacturing of micro and large glass components and will enable a wide variety of applications in science, industry, and daily life.

In order to help the readers select more suitable printing technologies and printing materials for glass 3D printing, a summary of the possible printing technologies and materials for 3D printing glass is given in Table 2.

## 4. Difficulties and Solutions for 3D Printing Glass Technology

The method of additive manufacturing has been gradually maturing and has great value in many fields. However, in the specific application of glass preparation through photopolymerization, it is found that the method still faces some challenges. These challenges are as followed: (1) the limitation of the ratio of photopolymerization resin, (2) the agglomeration of nanoparticles in “liquid glass” and the viscosity of the system, and (3) the inherent constraints of printing speed and printing resolution. To achieve better printing precision and faster printing speed, it is necessary to obtain the scientific proportioning of photocured resins and the uniform dispersion of nanopowders. To reach this goal, the related methods are reviewed in the following.

### 4.1. The Selection and Ratio of “Liquid Glass” and “Inks”

The core material of the above-mentioned technologies based on photopolymerization is “liquid glass”, which must meet two basic requirements when used in the printing process: good light-curing ability and fluidity [27,94]. Good photocuring ability is the basis for printing. Since photocuring systems lack the material distribution equipment of the SLS process, good flowability is an important property to ensure that the liquid photopolymer can be uniformly distributed over the printable area. In addition to the basic photopolymer and initiator, other additives are often used as important components to improve the print quality, including diluents, free radical inhibitors, and light absorbents. Diluents reduce liquid viscosity, free radical inhibitors prevent the premature gelling of acrylates, photoinitiators initiate monomer polymerization, and light absorbents inhibit the depth of cure. To keep the system stable for a reasonable time (e.g., from hours to days) without significant particle separation, a moderate amount of stabilizer is often required [2].

Because of the requirements for the degree of polymerization, the selection of polymeric monomers still has many areas to pay attention to. Firstly, having low viscosity and good dilution are the basic conditions. Secondly, the selected polymeric monomer should be able to dissolve a large amount of SiO_2_. Wozniak suggested that the polymeric monomer species can be found by using refractive index matching [95,96].

Inks suitable for DIW must have significant viscoelasticity to ensure their flow through the nozzle, must be able to maintain their shape during deposition, and should have shear thinning properties for smooth extrusion [97,98,99]. Viscoelasticity is particularly critical to avoid the collapse of structures draped through gaps, and elemental additions, such as GeO_2_ and PDMS, can be made to the ink to ensure its viscosity. Table 2 summarizes the possible technologies and material ratios for “liquid glass” and “inks” in the literature for reference.

### 4.2. Viscosity Reduction Methods for “Liquid Glass”

The amount of SiO_2_ nanoparticle incorporation is an important parameter of “liquid glass”, as high incorporation results in lower linear shrinkage after sintering and higher production precision [15,36]. However, the higher content of SiO_2_ leads to the high viscosity of the “liquid glass” system, which is not conducive to 3D printing; a lower content of SiO_2_ is good for printing but leads to the collapse and cracking of the molded object, and this contradiction is the main problem that limits the development of silica nano resin blends [36].

**Figure 7:**
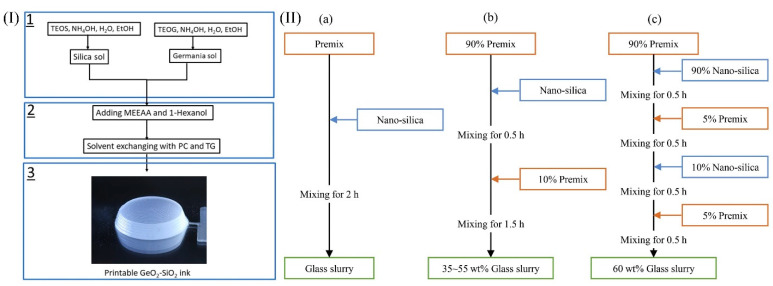
Viscosity reduction methods for “liquid glass”. (**I**) Reprinted with permission from ref. [15]. (**II**) Reprinted with permission from ref. [36].

To reduce the viscosity of liquid glass, Koroush Sasan et al. prepared a precursor slurry containing a mixture of inorganic precursors of alcohol salts and photosensitive monomers using the sol-gel method [15]. Replacing agglomerate-prone particle inks with liquid molecular precursors as the feedstock resin for 3D printing can effectively reduce the viscosity of the “liquid glass” system and produce a higher print resolution. The preparation process of the slurry is demonstrated in Figure 7I. The slurry could also be dried and sintered to obtain transparent glass devices, but due to the low solids loading, it resulted in a linear shrinkage of 33–56% and exhibited non-isotropic shrinkage and a low preparation efficiency of up to 7 days for the whole process.

To address the shortcomings of the sol-gel method, Peng Cai et al. investigated the effects of the slurry mixing method and solid content on the rheology of “liquid glass” and concluded that the viscosity of the multi-step mixing method was less than that of the one-step mixing method at different solid contents [36]. When the solid content of the slurry was 60 wt%, the viscosity of the multi-step mixing method and the one-step mixing method was 2059.7 mPa s and 5461.2 mPa s, respectively, at a shear rate of 0.1 s^−1^, with a difference of 3401.5 mPa s. The diagram of the slurry preparation is presented in Figure 7II. This also implies that the viscosity of the slurry can be effectively reduced by using a multi-step mixing method, which provides a new idea in the field of photocured 3D printing.

### 4.3. Methods to Improve the Precision and Speed of 3D Printing Glass

The inherent printing speed and factors affecting printing precision, such as the “step effect” of 3D printing, severely restrict the development of 3D-printed glass. These problems need to be solved, but few optimization solutions for 3D printing glass technology have been reported. Optimization solutions for polymer 3D printing can guide the optimization of 3D printing glass technology.

**Figure 8:**
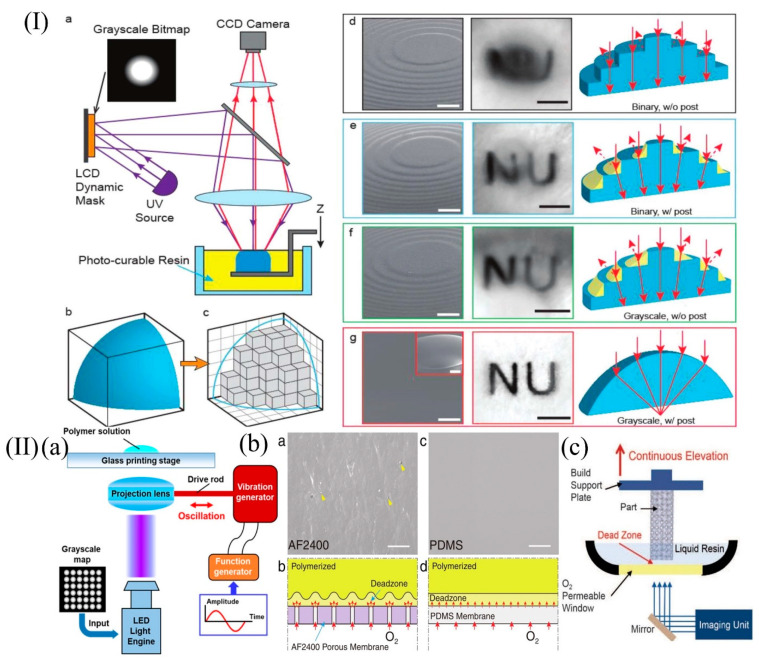
(**I**) 3D printing of an optically smooth surface using the PμSL system and post-processing steps. Panel (**a**), schematic illustration of the PμSL system. A 3D solid model with smooth surfaces shown in panel (**b**) is approximated as a set of discrete voxels; results in the pixelated rough surface are shown in panel (**c**). Panels (**d**–**g**), progressive improvement of surface roughness and the resulting imaging characteristic of 3D-printed lenses using various methods. (**II**) (**a**) Schematic of the oscillation-assisted DLP-based printing system. (**b**) Magnified SEM image shows in panel (**a**), an AF2400 Teflon membrane with distinct surface texture and pores and in panel (**c**), a PDMS membrane with smooth surface. Panel (**b**), schematic diagram of the different dead zone interfaces formed through a porous Teflon membrane with nonuniform oxygen permeability and in panel (**d**), a PDMS membrane with uniform oxygen permeability. (**I**) Reprinted with permission from ref. [100]. (**II**) (**a**) Reprinted with permission from ref. [101]. (**II**) (**b**) Reprinted with permission from ref. [21]. (**II**) (**c**) Reprinted with permission from ref. [102].

The most effective way to eliminate the step effect is to use grayscale exposure, combined with certain processing techniques, to obtain the best results. For example, in 2018, Xiangfan Chen et al. employed the projection micro-stereolithography process, and the synergistic effects from grayscale photopolymerization, alongside the meniscus equilibrium post-curing methods, helped to realize a high-speed 3D-printing process with subvoxel-scale precision (sub 5 μm) and deep subwavelength (sub 7 nm) surface roughness. The schematic diagram and the process of optimization are illustrated in Figure 8I [100]. In 2019, Chao Yuan et al. integrated DLP 3D printing with mechanical oscillation and grayscale UV exposure. The surface roughness was reduced from 200 nm to about 1 nm. The schematic of the principle is presented in Figure 8II(a) [101].

Surface roughness is also one of the important parameters affecting optical performance. To reduce the surface roughness of printed objects, in 2020, Sun Cheng et al. changed the direction of the micro-stereoscopic projection light to form a bottom-up projection method and replaced the Teflon oxygen permeable membrane with a PDMS to obtain a roughness of 13.7 nm resin single lens using only grayscale exposure. The mechanism analysis of surface roughness change is shown in Figure 8II(b) [21].

In addition, printing speed is a limiting factor for 3D printing technology. In 2015, John R et al. broke the limitations of the basic layer-by-layer deposition mechanism of SLA by proposing a new technique called continuous liquid interface production (CLIP). This technique enables the continuous curing of the resin, reduces the time for resin filling, and increases the printing efficiency to 500 mm^3^/h. The schematic of CLIP is presented in Figure 8II(c) [102].

In addition to the optimization methods mentioned above, appropriate post-processing can also be adopted to improve the quality of the object. Whether the post-processing is possible or not depends on which technology is chosen. Generally speaking, glass devices prepared using the SLA/DLP/TPP/SL processes don’t need post-processing, for they have a specific shape, and the shape determines the function. If the glasses printed with a specific shape are post-processed, these complex shapes will be destroyed and the function will be affected. However, post-processing is possible for DIW. For example, it has also been reported that polishing can also be used as a post-processing technique for the glasses that are fabricated using the DIW process to obtain the specific size and a surface microroughness of <1 nm root mean square for the glass devices to meet application requirements [14]. Moreover, to improve the preparation efficiency of the glass microstructure, in addition to shortening the time in the step of photopolymerization, the heat treatment process of reasonable optimization of 40–50 hours is often more significant than the step of polymerization forming. However, ideally, we must reasonably avoid problems, such as crimping cracks.

## 5. Perspective and Outlook

**Figure 9:**
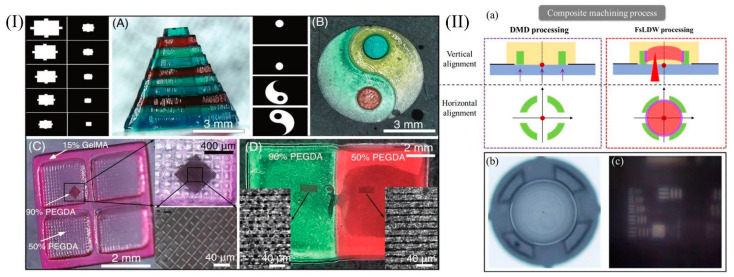
The structure processed using HLP technology is shown in (**A**–**D**) in (**I**). Samples prepared by our group using DMD and femtosecond laser composite processing technology are shown in (**II**). (**I**) Reprinted with permission from ref. [103].

In summary, glass additive manufacturing has considerable potential and could greatly contribute to academic research and industrial production. The next major trend in glass 3D printing technology is the composite processing of multiple technologies, with researchers intending to bring the advantages of multiple technologies together. However, there are few reports on composite processing for glass materials, and there are more reports on composite processing for polymers, which can be used as a theoretical guide for the composite processing of glass materials. For example, Pranav Soman’s team from Syracuse University has developed a novel hybrid laser printing (HLP) technique to print multi-scale, multi-material 3D hydrogel structures with a micron resolution through a process of continuous liquid forming (CLIP) combined with femtosecond laser multiphoton polymerization (MPP) and multiphoton ablation (MPA) [103]. As illustrated in Figure 9I(A), Mayan pyramids can be printed with 90% PEGDA, 1% LAP photoinitiator, and various food colorings; Figure 9I(B) illustrates a yin and yang structure printed using four different colors of PEGDA; Figure 9I(C) presents multiscale printing using two commonly used hydrogels (GelMA and PEGDA); in Figure 9I(D), additive and subtractive multi-material fabrication methods are shown. The inset shows an enlarged version of the ablation line using MPA.

Femtosecond laser-assisted DMD composite processing technology is also one of the recent research directions of our research group. On this basis, the hyperbolic lens prepared using an SU-8 photoresist is shown in Figure 9II(b). DMD and femtosecond laser direct writing techniques were used to process the support and lens parts, respectively. The maturity of this technology has greatly improved the efficiency of the structure preparation. The preliminary imaging effect is presented in Figure 9II(a–c) illustrate the composite machining process.

Due to the point-by-point scanning mechanism, the total fabrication time is proportional to ${\left(\frac{\mathrm{dimension}}{\mathrm{voxel}\;\mathrm{size}}\right)}^{3}$. Therefore, reducing the voxel dimensions will significantly slow down the 3D printing process. Reducing the voxel size by ten times will simply result in the 1000 times increase of the fabrication time [99]. Using DMD and femtosecond composite processing technology to process large and small parts of the object separately, and reducing the number of processing points to ensure printing precision, will greatly improve the efficiency of object preparation. The preparation of glass composite structures at different scales using composite processing techniques could also be applied in areas, such as hydrophobic surfaces. Femtosecond laser processing could also be used as a post-processing process in the 3D printing glass process by performing a secondary processing of the formed objects to compensate for the step effect and improve the precision of the objects.

In addition, the development of the optical field puts forward new requirements in which 3D printing glass technology is essential. Particularly, applying 3D printing to the optical realm would significantly improve the time-consuming and costly processes of fabricating customized optical elements, which result from the limitations of conventional methods, such as multiaxial lathes polishing, magnetorheological finishing, molding, and ion beam finishing techniques [104]. The rapid development of 3D printing glass optical components is needed to solve problems, such as the limitations of printing efficiency, face precision, and surface roughness. These limitations often determine the application area of the printed object. For example, if the precision and surface roughness can be adjusted to meet the requirements of optical components in the preparation process, high-performance optical components can be prepared. When the precision cannot meet the precision requirements of optical components, some micro and nanodevices with lower precision requirements, such as microfluidic chips, can also be prepared.

## 6. Conclusions

In summary, 3D printing technology, especially for glass materials, has emerged as a new application area with impressive progress. However, as an emerging technology, there are still many challenges that need to be solved before practical application, including, but not limited to, materials and design. In this review, 3D printing technologies for glass materials have been briefly introduced, and the current state of the main technologies used for manufacturing glass objects has been discussed in detail. Table 3 provides a thorough comparison of these technologies, listing the advantages and disadvantages of each aspect involved in the process. It is intended to help the reader by providing ideas for selecting the appropriate processing method to achieve high-speed and high-resolution printing.

## Figures and Tables

**Table 1:** Glass 3D printing technologies.

Categories	Glass 3D Printing Technology Type	Abbreviation
Powder based	Selective laser sintering	SLS
	Selective laser melting	SLM
Photopolymerization based	Stereolithography	SLA
	Two-photon polymerization	TPP
	Digital light processing	DLP
	Sheet lamination	SL
Material extrusion based	Direct ink writing	DIW
	Fused deposition modeling	FDM

**Table 2:** Some possible technologies and materials for 3D printing glass.

Technology	Polymerization of Monomer	Dispersant	Photoinitiator	Stabilizer	Absorber	SiO_2_	References
SLA	29.5 wt%HEMA ^1^+ 3.7 wt% PEGDA ^2^	—	0.4 wt% DMPA ^3^	13.1 wt% DEDB ^4^+ 0.4wt%MEHQ ^5^	0.3 wt% Tinuvin 1130	53.7 wt% Aerosil OX50	[39]
	28.1 wt%HEMA+3.7 wt% PEGDA	—	0.4 wt% DMPA	14.5 wt% DEDB	0.02 wt% Sudan red G	53.7 wt% Aerosil OX50	[76]
DLP	36.9 wt%HEMA+ 6.36 wt% TEGDA ^6^	19.0 wt% POE ^7^	0.2 wt% DPO ^8^	0.1 wt % hydroquinone	—	37.5wt% Aerosil OX50	[34]
	60 vol%HEMA+ 10 vol%TEGDA	30 vol% POE	0.2 wt% Omnirad 819 ^9^	0.1 wt% hydroquinone	0.05 wt% Sudan Orange G	37.5 vol% Aerosil OX50	[35]
	65 vol%HEMA+ 35 vol% TMPTA ^10^	—	0.5 wt% Omnirad 819	0.5 wt% hydroquinone	0.45 wt% Tinuvin 384-2	35 vol% Aerosil OX50	[35]
TPP	80 wt%HEMA	20 wt% POE	1 wt% BDK ^11^	—	—	50 wt% Aerosil OX50	[56]
	333 mg ETPTA ^12^+ 666 mgPEGDA	—	10 mg EMK ^13^	5mg hydroquinone	—	1600 mg colloidal silica solution	[57]
DIW	79 wt%Tetraglyme	—	—	1wt% PDMS	—	21 wt%CAB-O-SIL EH-5	[13]
	76 wt%Tetraglyme	—	—	1wt% PDMS	—	23 wt%CAB-O-SIL EH-5	[13]
SL	68 vol%HEMA+ 7 vol%TEGDA	25 vol% POE	0.5 wt% DMPA	—	—	40 vol% Aerosil OX50	[33]
	68 vol%HEMA+ 7 vol%TEGDA	25 vol% POE	0.5 wt% DMPA	—	—	50 vol% silica nanopowder (Zandosil)	[90]

^1^ HEMA: hydroxyethyl methacrylate. ^2^ PEGDA: poly(ethylene glycol) diacrylate. ^3^ DMPA: 2,2-dimethoxy-2-phenylacetophenone. ^4^ DEDB: diethyl phthalate. ^5^ MEHQ: 4-methoxyphenol. ^6^ TEGDA: tetraethylene glycol diacrylate. ^7^ POE: phenoxyethanol. ^8^ DPO: diphenyl(2,4,6-trimethylbenzoyl) phosphine oxide. ^9^ Omnirad 819: phenylbis(2,4,6-trimethylbenzoyl)phosphine oxide. ^10^ TMPTA: trimethylolpropane ethoxylate triacrylate. ^11^ BDK: photoinitiator (2,2-Dimethoxy-2-phenylacetophenone). ^12^ ETPTA: trimethylolpropane ethoxylate triacrylate. ^13^ EMK: 4,4′-Bis(diethylamino)benzophenone.

**Table 3:** Comparison of the different 3D printing technologies used for glass fabrication.

Technology	Power Source	Forming Method	Resolution	Component Size	Speed	Characteristic
SLA	Laser	Photopolymerization	μm	100 μm–100 mm	Slow	Precision molding
TPP	Laser	Photopolymerization	nm–μm	1 μm–1 mm	Slow	Ultra-high precision
DLP	Laser	Photopolymerization	μm	10 μm–100 mm	Medium	Rapid prototyping and high precision
DIW	Thermal energy	Extrusion	μm–mm	1 mm–10 cm	Medium	Muti-material molding
SL	Laser	Photopolymerization	μm	10 μm–100 cm	Fast	Large-size molding

## References

[1] Frazier W.E. (2014). Metal additive manufacturing: A review. J. Mater. Eng. Preform..

[2] Chen Z., Li Z., Li J., Liu C., Fu Y., Liu C., Li Y., Wang P., He Y., Lao C. (2019). 3D printing of ceramics: A review. J. Eur. Ceram. Soc..

[3] Huang S.H., Liu P., Mokasdar A., Hou L. (2013). Additive manufacturing and its societal impact: A literature review. Int. J. Adv. Manuf. Technol..

[4] Ligon S.C., Liska R., Stampfl J., Gurr M., Mülhaupt R. (2017). Polymers for 3D printing and customized additive manufacturing. Chem. Rev..

[5] Martin J.H., Yahata B.D., Hundley J.M., Mayer A.J., Schaedler T.A., Pollock T.M. (2017). 3D printing of high-strength aluminium alloys. Nature.

[6] Hirt L., Reiser A., Spolenak R., Zambelli T. (2017). Additive manufacturing of metal structures at the micrometer scale. Adv. Mater..

[7] Truby R.L., Lewis J.A. (2016). Printing soft matter in three dimensions. Nature.

[8] Wang D., Zhang J., Liu Q., Chen B., Liang Y., Hu L., Jiang G. (2020). 3D printing challenges in enabling rapid response to public health emergencies. Innovation.

[9] Wang X., Jiang M., Zhou Z., Gou J., Hui D. (2017). 3D printing of polymer matrix composites: A review and prospective. Compos. Part B-Eng..

[10] Yap C.Y., Chua C.K., Dong Z.L., Liu Z.H., Zhang D.Q., Loh L.E., Sing S.L. (2015). Review of selective laser melting: Materials and applications. Appl. Phys. Rev..

[11] Thiele S., Arzenbacher K., Gissibl T., Giessen H., Herkommer A. (2017). 3D-printed eagle eye: Compound microlens system for foveated imaging. Sci. Adv..

[12] Thomas R., Li J., Ladak S., Barrow D., Smowton P.M. (2018). In situ fabricated 3D micro-lenses for photonic integrated circuits. Opt. Express.

[13] Nguyen D.T., Meyers C., Yee T.D., Dudukovic N.A., Destino J.F., Zhu C., Duoss E.B., Baumann T.F., Suratwala T., Smay J.E. (2017). 3D-printed transparent glass. Adv. Mater..

[14] Dylla-Spears R., Yee T.D., Sasan K., Nguyen D.T., Dudukovic N.A., Ortega J.M., Johnson M.A., Herrera O.D., Ryerson F.J., Wong L.L. (2020). 3D printed gradient index glass optics. Sci. Adv..

[15] Sasan K., Lange A., Yee T.D., Dudukovic N., Nguyen D.T., Johnson M.A., Herrera O.D., Yoo J.H., Sawvel A.M., Ellis M.E. (2020). Additive manufacturing of optical quality germania–silica glasses. Acs. Appl. Mater. Interfaces.

[16] Zhang D., Xiao W., Liu C., Liu X., Ren J., Xu B., Qiu J. (2020). Highly efficient phosphor-glass composites by pressureless sintering. Nat. Commun..

[17] Wang J., Zheng B., Wang P. (2020). 3D printed Er^3+^/Yb^3+^ co-doped phosphosilicate glass based on sol-gel technology. J. Non-Cryst. Solids.

[18] Wang P., Chu W., Li W., Tan Y., Liu F., Wang M., Qi J., Lin J., Zhang F., Cheng Y. (2019). Three-Dimensional Laser Printing of Macro-Scale Glass Objects at a Micro-Scale Resolution. Micromachines.

[19] Hong Z., Ye P., Loy D.A., Liang R. (2021). Three-dimensional printing of glass micro-optics. Optica.

[20] Zhang Q., Lei J., Chen Y., Wu Y. (2019). 3D Printing of All-Glass Fiber-Optic Pressure Sensor for High Temperature Applications. IEEE Sens. J..

[21] Shao G., Hai R., Sun C. (2020). 3D printing customized optical lens in minutes. Adv. Opt. Mater..

[22] Wong K.V., Hernandez A. (2012). A review of additive manufacturing. ISRN Mech. Eng..

[23] Huang J., Qin Q., Wang J. (2020). A review of stereolithography: Processes and systems. Processes.

[24] Zhou L.Y., Fu J., He Y. (2020). A review of 3D printing technologies for soft polymer materials. Adv. Funct. Mater..

[25] Wang Y., John G., Pawlowski M.E., Tkaczyk T.S. (2018). 3D printed fiber optic faceplates by custom controlled fused deposition modeling. Opt. Express.

[26] Klein J., Stern M., Franchin G., Kayser M., Inamura C., Dave S., Weaver J., Houk P., Colombo P., Yang M. (2015). Additive manufacturing of optically transparent glass. 3D Print. Addit. Manuf..

[27] Baudet E., Ledemi Y., Larochelle P., Morency S., Messaddeq Y. (2019). 3D-printing of arsenic sulfide chalcogenide glasses. Opt. Mater. Express.

[28] Luo J., Edward H.P., Kinzel C. (2014). Additive manufacturing of glass. J. Manuf. Sci. Eng..

[29] Luo J., Hostetler J.M., Gilbert L., Goldstein J.T., Urbas A.M., Bristow D.A., Landers R.G., Kinzel E.C. (2018). Additive manufacturing of transparent fused quartz. Opt. Eng..

[30] Luo J., Gilbert L.J., Qu C., Robert G.L., Douglas A.B., Edward C.K. (2017). Additive manufacturing of optically transparent soda-lime glass using a filament-fed process. J. Manuf. Sci. Eng..

[31] Kotz F., Schneider N., Striegel A., Wolfschläger A., Keller N., Worgull M., Bauer W., Schild D., Milich M., Greiner C. (2018). Glassomer—processing fused silica glass like a polymer. Adv. Mater..

[32] Layani M., Wang X., Magdassi S. (2018). Novel materials for 3D printing by photopolymerization. Adv. Mater..

[33] Kotz F., Plewa K., Bauer W., Schneider N., Keller N., Nargang T., Helmer D., Sachsenheimer K., Schäfer M., Worgull M. (2016). Liquid glass: A facile soft replication method for structuring glass. Adv. Mater..

[34] Chu Y., Fu X., Luo Y., Canning J., Tian Y., Cook K., Zhang J., Peng G. (2019). Silica optical fiber drawn from 3D printed preforms. Opt. Lett..

[35] Kotz F., Arnold K., Bauer W., Schild D., Keller N., Sachsenheimer K., Nargang T.M., Richter C., Helmer D., Rapp B.E. (2017). Three-dimensional printing of transparent fused silica glass. Nature.

[36] Cai P., Guo L., Wang H., Li J., Li J., Qiu Y., Zhang Q., Lue Q. (2020). Effects of slurry mixing methods and solid loading on 3D printed silica glass parts based on DLP stereolithography. Ceram. Int..

[37] Cooperstein I., Shukrun E., Press O., Kamyshny A., Magdassi S. (2018). Additive manufacturing of transparent silica glass from solutions. ACS Appl. Mater. Interfaces.

[38] Gal-Or E., Gershoni Y., Scotti G., Nilsson S.M.E., Saarinen J., Jokinen V., Strachan C.J., Gennäs G.B., Yli-Kauhaluoma J., Kotiaho T. (2019). Chemical analysis using 3D printed glass microfluidics. Anal. Methods.

[39] Liu C., Qian B., Liu X., Tong L., Qiu J. (2018). Additive manufacturing of silica glass using laser stereolithography with a top-down approach and fast debinding. RSC Adv..

[40] Maruo S., Fourkas J.T. (2008). Recent progress in multiphoton microfabrication. Laser Photonics Rev..

[41] Melchels F.P.W., Feijen J., Grijpma D.W. (2010). A review on stereolithography and its applications in biomedical engineering. Biomaterials.

[42] Jariwala S.H., Lewis G.S., Bushman Z.J., Adair J.H., Donahue H.J. (2015). 3D printing of personalized artificial bone scaffolds. 3D Print Addit. Manuf..

[43] Pham D.T., Gault R.S. (1998). A comparison of rapid prototyping technologies. Int. J. Mach. Tools Manuf..

[44] Oropallo W., Piegl L.A. (2016). Ten challenges in 3D printing. Eng. Comput..

[45] Rengier F., Mehndiratta A., Tengg-Kobligk H.V., Zechmann C.M., Unterhinninghofen R., Kauczor H.U., Giesel F.L. (2010). 3D printing based on imaging data: Review of medical applications. Int. J. Comput. Assisted Radiol. Surg..

[46] Griffith M.L., Halloran J.W. (1996). Freeform fabrication of ceramics via stereolithography. J. Am. Ceram. Soc..

[47] Eckel Z.C., Zhou C., Martin J.H., Jacobsen A.J., Carter W.B., Schaedler T.A. (2016). Additive manufacturing of polymer-derived ceramics. Science.

[48] Brady G.A., Halloran J.W. (1997). Stereolithography of ceramic suspensions. Rapid Prototyp. J..

[49] Halloran J.W., Tomeckova V., Gentry S., Das S., Cilino P., Yuan D., Guo R., Rudraraju A., Shao P., Wu T. (2011). Photopolymerization of powder suspensions for shaping ceramics. J. Eur. Ceram. Soc..

[50] Bae C.J., Ramachandran A., Halloran J.W. (2018). Quantifying particle segregation in sequential layers fabricated by additive manufacturing. J. Eur. Ceram. Soc..

[51] Maruo S., Nakamura O., Kawata S. (1997). Three-dimensional microfabrication with two-photon-absorbed photopolymerization. Opt. Lett..

[52] Schizas C., Melissinaki V., Gaidukeviciute A., Reinhardt C., Ohrt C., Dedossis V., Chichkov B.N., Fotakis C., Farsari M., Karalekas D. (2010). On the design and fabrication by two-photon polymerization of a readily assembled micro-valve. Int. J. Adv. Manuf. Technol..

[53] Seet K.K., Mizeikis V., Matsuo S., Juodkazis S., Misawa H. (2005). Three-dimensional spiral-architecture photonic crystals obtained by direct laser writing. Adv. Mater..

[54] Kawata S., Sun H.B., Tanaka T., Takada K. (2001). Finer features for functional microdevices. Nature.

[55] Gan Z., Cao Y., Evans R.A., Gu M. (2012). Three-dimensional deep sub-diffraction optical beam lithography with 9 nm feature size. Nat. Commun..

[56] Doualle T., André J.C., Gallais L. (2021). 3D printing of silica glass through a multiphoton polymerization process. Opt. Lett..

[57] Wen X., Zhang B., Wang W., Ye F., Yue S., Guo H., Gao G., Zhao Y., Fang Q., Nguyen C. (2021). 3D-printed silica with nanoscale resolution. Nat. Mater..

[58] Wu D., Zhao Z., Zhang Q., Qi H.J., Fang D. (2019). Mechanics of shape distortion of DLP 3D printed structures during UV post-curing. Soft Matter.

[59] Komissarenko D.A., Sokolov P.S., Evstigneeva A.D., Shmeleva I.A., Dosovitsky A.E. (2018). Rheological and curing behavior of acrylate-based suspensions for the DLP 3D printing of complex zirconia parts. Materials.

[60] Nakamoto T., Yamaguchi K. Consideration on the producing of high aspect ratio micro parts using UV sensitive photopolymer. Proceedings of the 7th International Symposium on Micro Machine and Human Science.

[61] Son J.Y., Lee B.R., Chernyshov O.O., Moon K.A., Lee H. (2013). Holographic display based on a spatial DMD array. Opt. Lett..

[62] Ri S., Fujigaki M., Matui T., Morimoto Y. (2006). Pixel-to-pixel correspondence adjustment in DMD camera by moiré methodology. Exp. Mech..

[63] Sun C., Fang N., Wu D., Zhang X. (2005). Projection micro-stereolithography using digital micro-mirror dynamic mask. Sens. Actuators A Phys..

[64] Sadeqi A., Nejad R.H., Owyeung R.E., Sonkusale S. (2019). Three dimensional priting of metamaterial embedded geometrical optics (MEGO). Microsyst. Nanoeng..

[65] Waheed S., Cabot J.M., Macdonald N.P., Lewis T., Guijt R.M., Paull B., Breadmore M.C. (2016). 3D printed microfluidic devices: Enablers and barriers. Lab Chip.

[66] Manapat J.Z., Chen Q., Ye P., Advincula R.C. (2017). 3D printing of polymer nanocomposites via stereolithography. Macromol. Mater. Eng..

[67] Sharafeldin M., Kadimisetty K., Bhalerao K.S., Chen T., Rusling J.F. (2020). 3D-printed Immunosensor arrays for cancer diagnostics. Sensors.

[68] Nan B., Galindo-Rosales F.J., Ferreira J.M.F. (2020). 3D printing vertically: Direct ink writing free-standing pillar arrays. Mater. Today.

[69] Zhu J., Zhang Q., Yang T., Liu Y., Liu R. (2020). 3D printing of multi-scalable structures via high penetration near-infrared photopolymerization. Nat. Commun..

[70] Kim Y., Yuk H., Zhao R., Chester S.A., Zhao X. (2018). Printing ferromagnetic domains for untethered fast-transforming soft materials. Nature.

[71] Hardin J.O., Ober T.J., Valentine A.D., Lewis J.A. (2015). Microfluidic printheads for multimaterial 3D printing of viscoelastic inks. Adv. Mater..

[72] Kowsari K., Akbari S., Wang D., Fang N.X., Ge Q. (2018). High-efficiency high-resolution multimaterial fabrication for digital light processing-based three-dimensional printing. 3D Print. Addit. Manuf..

[73] Ma X., Qu X., Zhu W., Li Y.-S., Yuan S., Zhang H., Liu J., Wang P., Lai C.S.E., Zanella F. (2016). Deterministically patterned biomimetic human iPSC-derived hepatic model via rapid 3D bioprinting. Proc. Natl. Acad. Sci. USA.

[74] Destino J.F., Dudukovic N.A., Johnson M.A., Nguyen D.T., Yee T.D., Egan G.C., Sawvel A.M., Steele W.A., Baumann T.F., Duoss E.B. (2018). 3D printed optical quality silica and silica-titania glasses from sol-gel feedstocks. Adv. Mater. Technol..

[75] Beh W.S., Kim I.T., Qin D., Xia Y., Whitesides G.M. (1999). Formation of patterned microstructures of conducting polymers by soft lithography, and applications in microelectronic device fabrication. Adv. Mater..

[76] Liu C., Qian B., Ni R., Liu X., Qiu J. (2018). 3D printing of multicolor luminescent glass. RSC Adv..

[77] Zhang A.P., Qu X., Soman P., Hribar K.C., Lee J.W., Chen S., He S. (2012). Rapid fabrication of complex 3D extracellular microenvironments by dynamic optical projection stereolithography. Adv. Mater..

[78] Lee M.P., Cooper G.J., Hinkley T., Gibson G.M., Padgett M.J., Cronin L. (2015). Development of a 3D printer using scanning projection stereolithography. Sci. Rep..

[79] Zhang D., Liu X., Qiu J. (2021). 3D printing of glass by additive manufacturing techniques: A review. Front. Optoelectron..

[80] Moore D.G., Barbera L., Masania K., Studart A.R. (2020). Three-dimensional printing of multicomponent glasses using phase-separating resins. Nat. Mater..

[81] Kotz F., Risch P., Arnold K., Sevim S., Puigmartí-Luis J., Quick A., Thiel M., Hrynevich A., Dalton P.D., Helmer D. (2019). Fabrication of arbitrary three-dimensional suspended hollow microstructures in transparent fused silica glass. Nat. Commun..

[82] Helmer D., Voigt A., Wagner S., Keller N., Sachsenheimer K., Kotz F., Nargang T.M., Rapp B.E. (2017). Suspended liquid subtractive lithography: One-step generation of 3D channel geometries in viscous curable polymer matrices. Sci. Rep..

[83] Saggiomo V., Velders A.H. (2015). Simple 3D printed scaffold-removal method for the fabrication of intricate microfluidic devices. Adv. Sci..

[84] Patrick J.F., Krull B.P., Garg M., Mangun C.L., Moore J.S., Sottos N.R., White S.R. (2017). Robust sacrificial polymer templates for 3D interconnected microvasculature in fiber-reinforced composites. Compos. Part A Appl. Sci. Manuf..

[85] Hedayat N., Du Y., Ilkhani H. (2017). Review on fabrication techniques for porous electrodes of solid oxide fuel cells by sacrificial template methods. Renew. Sustain. Energy Rev..

[86] Gong H., Bickham B.P., Woolley A.T., Nordin G.P. (2017). Custom 3D printer and resin for 18 μm × 20 μm microfluidic flow channels. Lab Chip.

[87] Wang W., Tafti G., Ding M., Luo Y., Tian Y., Wang S., Karpisz T., Canning J., Cook K., Peng G.D. (2018). Structure formation dynamics in drawing silica photonic crystal fibres. Front. Optoelectron..

[88] Waldbaur A., Carneiro B., Hettich P., Wilhelm E., Rapp B.E. (2013). Computer-aided microfluidics (CAMF): From digital 3D-CAD models to physical structures within a day. Microfluid. Nanofluid..

[89] Martelli C., Canning J. (2007). Fresnel fibres with omnidirectional zone cross-sections. Opt. Express.

[90] Langenhorst M., Ritzer D., Kotz F., Risch P., Dottermusch S., Roslizar A., Schmager R., Richards B.S., Rapp B.E. (2019). Liquid Glass for Photovoltaics: Multifunctional Front Cover Glass for Solar Modules. ACS Appl. Mater. Interfaces.

[91] Li X.M., Reinhoudt D., Crego-Calama M. (2007). What Do We Need for a Superhydrophobic Surface? A Review on the Recent Progress in the Preparation of Superhydrophobic Surfaces. Chem. Soc. Rev..

[92] Wang R., Hashimoto K., Fujishima A., Chikuni M.M., Kojima E., Kitamura A., Shimohigoshi M., Watanabe T., Wilbur J.L., Biebuyck H.A. (1997). Light-Induced Amphiphilic Surfaces. Nature.

[93] Roslizar A., Dottermusch S., Vüllers F., Kavalenka M.N., Guttmann M., Schneider M., Paetzold U.W., Hölscher H., Richards B.S., Klampaftis E. (2019). Self-Cleaning Performance of Superhydrophobic Hot-Embossed Fluoropolymer Films for Photo-voltaic Modules. Sol. Energy Mater. Sol. Cells.

[94] Zhang Y., Dong Z., Li C., Du H., Fang N., Wu L., Song Y. (2020). Continuous 3D printing from one single droplet. Nat. Commun..

[95] Wozniak M., Graule T., de Hazan Y., Kata D., Lis J. (2009). Highly loaded UV curable nanosilica dispersions for rapid prototyping applications. J. Eur. Ceram. Soc..

[96] Wozniak M., de Hazan Y., Graule T., Kata D. (2011). Rheology of UV curable colloidal silica dispersions for rapid prototyping applications. J. Eur. Ceram. Soc..

[97] Lewis J.A. (2006). Direct ink writing of 3D functional materials. Adv. Funct. Mater..

[98] Zhang Z., Jin Y., Yin J., Xu C., Xiong R., Christensen K., Ringeisen B.R., Chrisey D.B., Huang Y. (2018). Evaluation of bioink printability for bioprinting applications. Appl. Phys. Rev..

[99] Paxton N., Smolan W., Böck T., Melchels F., Groll J., Jungst T. (2017). Proposal to assess printability of bioinks for extrusion-based bioprinting and evaluation of rheological properties governing bioprintability. Biofabrication.

[100] Chen X., Liu W., Dong B., Lee J., Ware H.O.T., Zhang H.F., Sun C. (2018). High-Speed 3D Printing of Millimeter-Size Customized Aspheric Imaging Lenses with Sub 7 nm Surface Roughness. Adv. Mater..

[101] Yuan C., Kowsari K., Panjwani S., Chen Z., Wang D., Zhang B., Ng C.J.-X., Valdivia y Alvarado P.V., Ge Q. (2019). Ultrafast three-dimensional printing of optically smooth microlens arrays by oscillation-assisted digital light processing. ACS Appl. Mater. Interfaces.

[102] Tumbleston J.R., Shirvanyants D., Ermoshkin N., Janusziewicz R., Johnson A.R., Kelly D., Chen K., Pinschmidt R., Rolland J.P., Ermoshkin A. (2015). Continuous liquid interface production of 3D objects. Science.

[103] Kunwar P., Xiong Z., Zhu Y., Li H., Filip A., Soman P. (2019). Hybrid laser printing of 3D, multiscale, multimaterial hydrogel structures. Adv. Opt. Mater..

[104] Gissibl T., Thiele S., Herkommer A., Giessen H. (2016). Sub-micrometre accurate free-form optics by three-dimensional printing on single-mode fibres. Nat. Commun..

